# Massage Establishments

**Published:** 1920-05-15

**Authors:** 


### MASSAGE ESTABLISHMENTS.

### Powers Asked for to Remedy Abuses.

The London County Council is taking steps to obtain powers by which it hopes to regulate existing abuses in regard to massage establishments. At present these establishments are held under a system of registration, but the Council hopes, by substituting for that a system of licensing, to gain a more satisfactory power control. The Select Committee of the House of Commons, which is considering the Bill that includes this provision, was informed by representatives and officers of the Council that the legislation passed in 1915 is inadequate, and not nearly strong enough to enable the authorities to suppress a grave social evil which has been shown to exist. Under that legislation the Council has so much to do before it can revise registration or strike a person off the register, that little has been done to cope with abuses. Licensing sessions are held in public, and the moral effect of an annual review of the licensee's conduct is very important. Under registration the moral effect of publicity is lacking, and, as one of the officials phrased it, the Council's "hands are tied behind its back." The Council also asks for new grounds upon which licences may be refused, such as unsuitability of premises, the unsatisfactory conduct of the business, or the possession of inadequate technical qualifications of the personnel.

Some instances were given to the Committee of the need for such provisions. It was stated, for example, that there is a woman on the register in respect of a massage establishment. She is carrying on business on behalf of a man who was refused registration—a man who was undoubtedly, in the early days of massage establishments, well known as a bad lot. "We cannot prove that the woman herself is of doubtful character," stated the chief officer of the Public Control Department of the Council. "We cannot bring proof against her in such a way as to substantiate the charge that she is a woman of immoral conduct, and yet the whole history of the woman is sufficient to indicate that she is not a proper woman to carry on a massage establishment. Then there are women in several of these establishments who are improperly dressed. There are women who are known to associate with prostitutes or other bad characters. We cannot bring a charge against them. There are persons who, after being registered, exercise no supervision over the establishment, but leave the management entirely to young women assistants. There are women who advertise their registered establishments in a certain way, namely, as English, French, or Scottish masseuses, or give Christian names in order to attract people to the establishments who do not want to go there for purposes of massage: frequently the assistants are not paid a proper wage, but depend almost entirely for their remuneration on the tips they receive. Nearly all the clients are men, and we know from the way in which the girls dress and where their evenings are passed that they must spend a considerable amount of money, which, obviously, they do not receive from their occupation at massage work."

In many massage establishments the assistants are not properly qualified, and it is desirable that the assistants should be qualified persons.

There are establishments that may be owned by a man or woman not necessarily skilled in massage work, but dependent upon assistants, who should be properly skilled. It was contended that the opportunities for training are sufficient for those desirous of entering the profession.

The Committee agreed to the clauses, and it is to be hoped that, when the Council eventually enjoys the powers which the clauses convey, it will be able to remedy abuses. The condition of things outlined is certainly scandalous, and, to put it on no higher grounds, it is only just to the properly conducted massage establishments that the less reputable places should be ruthlessly weeded out.

